# Decomposition Mechanisms and Kinetics of Novel Energetic Molecules BNFF-1 and ANFF-1: Quantum-Chemical Modeling

**DOI:** 10.3390/molecules18078500

**Published:** 2013-07-18

**Authors:** Roman V. Tsyshevsky, Maija M. Kuklja

**Affiliations:** Materials Science and Engineering Department, University of Maryland, College Park, MD 20742, USA; E-Mail: rtsyshev@umd.edu

**Keywords:** molecular materials, high explosives, LLM-175, LLM-172, heterocycles, activation barrier and transition state, density functional theory

## Abstract

Decomposition mechanisms, activation barriers, Arrhenius parameters, and reaction kinetics of the novel explosive compounds, 3,4-bis(4-nitro-1,2,5-oxadiazol-3-yl)-1,2,5-oxadiazole (BNFF-1), and 3-(4-amino-1,2,5-oxadiazol-3-yl)-4-(4-nitro-1,2,5-oxadiazol-3-yl)-1,2,5-oxadiazole (ANFF-1) were explored by means of density functional theory with a range of functionals combined with variational transition state theory. BNFF-1 and ANFF-1 were recently suggested to be good candidates for insensitive high energy density materials. Our modeling reveals that the decomposition initiation in both BNFF-1 and ANFF-1 molecules is triggered by ring cleavage reactions while the further process is defined by a competition between two major pathways, the fast C-NO_2_ homolysis and slow nitro-nitrite isomerization releasing NO. We discuss insights on design of new energetic materials with targeted properties gained from our modeling.

## 1. Introduction

Explosive decomposition consists of three main stages: initiation, combustion, and detonation. The two latter aspects are fairly well understood especially on the macro- and meso-scales. By contrast, a detailed understanding of the initiation process has yet to be established. It is clear that the initiation of chemistry in energetic compounds is a complex process, and hence progress in searching for new and improved energetic compounds has been rather slow. With recent advances in both computational capabilities [1,2] and ultra-fast time resolved spectroscopic (optical) diagnostics [3], attention has begun to focus on the characterization of the physicochemical responses of specific energetic ingredients such as localized mechanical stresses [4,5,6,7,8], crystal packing [9,10,11], morphology [12,13], and details of chemical decomposition reactions [14,15].

It is accepted that sensitivity to initiation of detonation (or a propensity of an energetic material to trigger a chemical reaction of explosive decomposition) depends upon many factors including chemical composition, bonding of functional groups, distribution of the charge density, stoichiometry, molecular ordering, grain size, presence of imperfections, nanoparticles, interfaces, *etc.* However, correlations between the structure, composition, and sensitivity have yet to be revealed. Fundamental insights into these relationships are essential so that energetic materials’ properties can be tailored to influence macroscopic responses. Understanding factors that govern sensitivity and at the same time provide high performance represents an outstanding challenge. Making inroads into these questions is the goal of our study.

**Figure 1:**
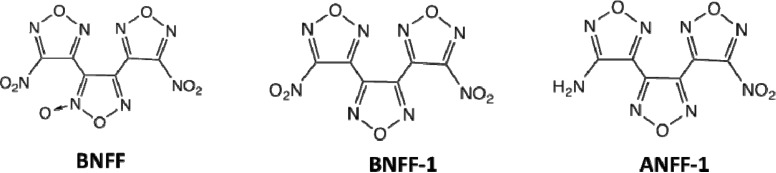
Structures of 3,4-bis(4-nitro-1,2,5-oxadiazol-3-yl)-1,2,5-oxadiazole-N-oxide (BNFF), 3,4-bis(4-nitro-1,2,5-oxadiazol-3-yl)-1,2,5-oxadiazole (BNFF-1) and 3-(4-amino-1,2,5-oxadiazol-3-yl)-4-(4-nitro-1,2,5-oxadiazol-3-yl)-1,2,5-oxadiazole (ANFF-1).

3,4-bis(4-Nitro-1,2,5-oxadiazol-3-yl)-1,2,5-oxadiazole-*N*-oxide (BNFF, also referred to as DNTF) has received considerable attention due to its good thermal stability, low melting point (110 °C), high density (1.937g/cm^3^), and moderate sensitivity [16,17,18,19,20,21]. Newly synthesized BNFF analogs, 3,4-bis(4-nitro-1,2,5-oxadiazol-3-yl)-1,2,5-oxadiazole (BNFF-1, also known as LLM-172) and 3-(4-amino-1,2,5-oxadiazol-3-yl)-4-(4-nitro-1,2,5-oxadiazol-3-yl)-1,2,5-oxadiazole (ANFF-1, also known as LLM-176), were recently suggested to be good candidates for insensitive high explosives (Figure 1). Low melting temperatures of BNFF-1 (85 °C) and ANFF-1 (100 °C) coupled with their predicted good stability in the melt make these materials attractive as melt-castable explosives. Forecasted distinctive physical and detonation properties of BNFF inspired a series of computational studies of thermodynamics and reactivity of various furazan derivatives [22,23,24,25] and optically excited furazan based molecules [26,27]. However, complexity of those molecules calls for sophisticated calculations to help with an interpretation of experimental predictions. For example, the thermal stability of furazan based compounds, evaluated by comparing homolytic bond cleavage energies alone [22] is fairly quick, but insufficient as the kinetics of the molecular decomposition mechanisms have to be taken into account to draw a definite conclusion. With the lack of an analysis of alternative primary steps besides a scission of the C-N bond between the furazan ring and the azoxy-group, a discussion of the decomposition mechanisms of two radical moieties left after a fragmentation of 3,3'-dinitro-4,4'-azoxyfurazan (DNOAF) [20] has a limited use. Theoretical studies provide a powerful tool to complement high quality experiments in order to advance our knowledge of the decomposition processes. Hence modeling of chemical reactions in particular materials should be performed as comprehensively as possible to obtain meaningful results and unambiguous conclusions. 

This article, aimed at gaining the first-principles fundamental understanding of intramolecular interactions and elucidation of the ground state thermal decomposition chemistry of novel nitro-substituted heterocyclic energetic molecules BNFF-1 and ANFF-1, reports several candidate decomposition mechanisms, the corresponding activation barriers, Arrhenius parameters, and reaction kinetics. Although materials response to thermal perturbation cannot be judged or determined based on molecular calculations alone and solid-state modeling is required, this study represents the first necessary step and provides useful insight into the decomposition reaction mechanisms of these novel molecules.

## 2. Computational Details

Calculations were performed using density functional theory (DFT) [28,29] with the PBE exchange correlation functional, [30] and hybrid B3LYP [31,32] and M06 [33] functionals, with double-zeta cc-pVDZ Dunning’s correlation consistent basis set [34] as implemented in the GAUSSIAN 09 code package [35]. PBE functional is widely used for investigating solid state reaction mechanisms [2] (it is implemented in most periodic computer codes because of its efficiency) and hence gas-phase calculations are required before any studies of decomposition pathways in practical materials are attempted (see, for example, ref. [4,5,6,13]). Unlike PBE, both hybrid B3LYP and M06 functionals consume significant time and computer resources but are usually considered relatively reliable for modeling reactions in gaseous phase (see, for example, ref. [36]). Vibrational frequencies have been calculated for relevant atomistic configurations to distinguish energy minima and transition states and to determine corresponding zero-point energy (ZPE) corrections. The stationary points corresponding to the energy minimum have been positively identified by having no imaginary frequencies and the transition states had exactly one imaginary frequency. An intrinsic reaction coordinate (IRC) analysis was carried out by using the Hessian-based Predictor-Corrector integrator algorithm [37,38] for each transition state to ensure that it was the transition structure, connecting the desired reactants and products. Pre-exponential factors were calculated with conventional transition state theory (TST) [39] for the decomposition reactions that proceed through a formation of a transition state and with variational transition state theory (VTST) [40] for the homolytic cleavage pathways, as detailed elsewhere [13,41,42,43]. 

## 3. Simulating Chemical Decomposition Reactions

**Figure 2:**
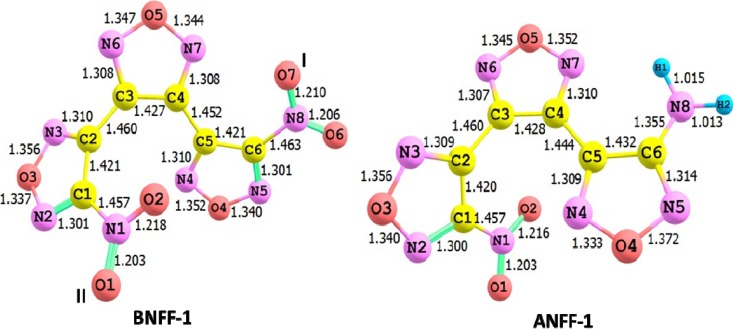
The geometry structures of BNFF-1 and ANFF-1. The non-equivalent nitro-groups in BNFF-1 are denoted with (I) and (II). The nitro-group (I) is substituted with an amino-group to form ANFF-1. The shown bond distances (Å) correspond to the M06 level of theory.

The geometric structures of the BNFF-1 and ANFF-1 molecules in equilibrium obtained with the M06 method are depicted in Figure 2. Both BNFF-1 and ANFF-1 molecules have fairly similar non-planar structures. The calculated equilibrium bond distances of the BNFF-1 molecule are very close to those of ANFF-1. Figure 2 demonstrates that a substitution of the nitro group in BNFF-1 by the amino group in ANFF-1 affects only the fragment connected to the nitro-group (the C5-C6-N5-O4-N4 ring and C4-C5 bond distances) and introduces negligible changes in the rest of the molecule. The C5-C6, C6-N5, N5-O4 bond distances in ANFF-1 are slightly elongated and O4-N4 and C4-C5 bond lengths are slightly decreased as compared to BNFF-1. The torsion angles φ(C4C3C2C1) and φ(C3C4C5C6) corresponding to a rotation of the ring fragments about C2-C3 and C4-C5 bonds in BNFF-1 are 51.3° and −162.9°, respectively. A nearly planar fragment (φ(C3C4C5C6) = −177.8°) of the ANFF-1 molecule consists of the central and amino-furazan rings; the nitro-furazan fragment is rotated about C2-C3 bond by 53.9°. All geometry parameters obtained within different DFT functionals employed agree well with each other with the average discrepancy between bond distances in ANFF-1 calculated at M06 and B3LYP does not exceed 0.6%. 

**Figure 3:**
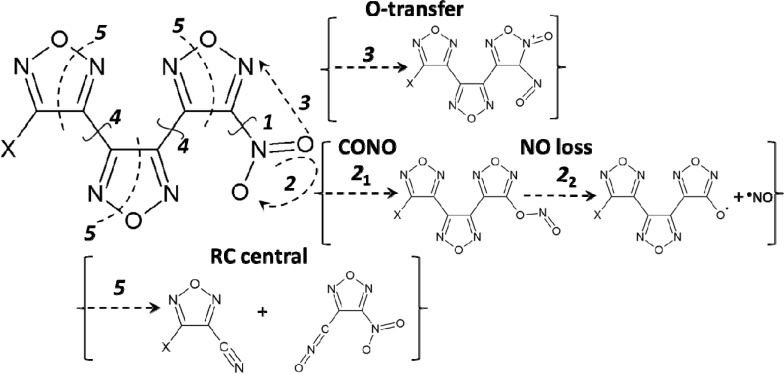
The sketched decomposition mechanisms represent five selected initiation reactions in BNFF-1 and ANFF-1. The X stands for a nitro-group in BNFF-1 and an amino-group in ANFF-1.

Thermal stability of the BNFF-1 and ANFF-1 molecules was explored through modeling the five most plausible decomposition mechanisms, schematically illustrated in Figure 3. The homolytic cleavage of the C-N bond (reaction 1, Table 1), leading to NO_2_ loss, is usually assumed to be the most favorable decomposition channel of nitro compounds. The two-step reaction of the nitro-nitrite rearrangement (CONO-isomer formation), proceeding via a pseudo rotation of the nitro group (reaction 2, Table 1), results in a release of NO and is often considered as an alternative primary dissociation reaction of nitro-arenes. The oxygen transfer involves the bond switching of the oxygen atom from the nitro group to the ring nitrogen (reaction 3, Table 1). We also probed the homolytic cleavage of the C-C bonds that connect two rings (reactions 4, Table 1) and a fragmentation of the molecules through the furazan ring cleavage (RC, reactions 5, Table 1).

**Table 1:** Calculated activation barriers E (kcal/mol), zero-point energy corrected barriers, E_ZPE_ (kcal/mol), and pre-exponential factors (log(A/^s−1^)) of BNFF-1 and ANFF-1 decomposition reactions.

Reaction		PBE			M06			B3LYP		
		E	E_ZPE_	logA	E	E_ZPE_	logA	E	E_ZPE_	logA
**BNFF-1 (LLM-172)**										
*1*	**NO_2_ loss (I) ^a^**	64.9	61.1	17.6	67.0	63.1	17.2	61.6	57.6	17.6
	**NO_2_ loss (II) ^a^**	66.3	62.2	18.1	69.7	65.6	17.9	64.3	60.0	18.2
*2*	**CONO (I)**	48.3	46.1 (−13.3) ^b^	13.2	55.4	53.2 (−12.8)	13.2	55.4	53.2 (−14.6)	13.2
	**CONO (II)**	50.2	47.9 (−11.6)	13.8	58.2	55.9 (−10.0)	14.0	-	-	-
	**NO loss (I)^c^**	34.1	30.9	-	27.9	25.0	-	22.8	19.8	-
	**NO loss (II)**	33.9	30.8	-	27.9	24.9	-	-	-	-
*3*	**O-transfer(I)**	71.6	69.4 (17.9)	12.9	82.3	80.3 (24.3)	12.7	80.1	77.9 (22.7)	12.6
	**O-transfer(II)**	74.8	72.4 (13.0)	13.0	86.8	84.6 (25.1)	12.5	84.3	81.9 (23.1)	12.7
*4*	**C-C (I)**	127.1	122.6	-	129.5	125.4	-	125.3	120.9	-
	**C-C (II)**	129.3	124.8	-	131.2	127.1	-	126.6	122.2	-
*5*	**RC central**	45.7	42.9 (27.6)	15.3	50.5	47.6 (27.3)	14.0	50.4	47.5 (23.3)	15.1
	**RC (I)**	48.3	45.4 (36.6)	14.9	53.7	50.7 (37.5)	14.0	54.0	50.8 (34.1)	14.8
	**RC (II)**	48.3	45.3 (36.4)	15.2	55.2	52.1 (38.0)	14.3	54.3	51.1	15.0
**ANFF-1 (LLM-175)**										
*1*	**NO_2_ loss**	65.4	61.3	18.1	65.2	61.5	18.0	63.8	59.5	18.3
*2*	**CONO**	54.3	51.9 (−9.8)	13.8	62.6	60.3 (−8.2)	13.7	61.6	59.1 (−10.7)	13.9
	**NO loss^c^**	32.9	29.8	-	26.8	23.8	-	21.6	18.6	-
*3*	**O-transfer**	77.0	74.6 (18.4)	13.4	88.6	88.5 (25.0)	12.9	85.8	83.5 (23.1)	13.1
*4*	**C-C (NO_2_)**	130.3	125.8	-	132.1	128.1	-	127.5	123.2	-
	**C-C (NH_2_)**	134.3	129.8	-	136.4	132.4	-	132.2	127.7	-
*5*	**RC central**	45.5	42.7 (27.5)	15.2	50.6	47.6 (24.6)	14.9	50.0	47.1 (23.8)	15.0
	**RC (NO_2_)**	48.5	45.5 (32.4)	15.2	54.9	51.8 (30.3)	14.2	54.4	51.3 (31.1)	15.1
	**RC (NH_2_)**	50.6	47.3 (40.6)	15.4	53.7	50.8 (39.7)	14.6	55.6	52.2 (36.3)	15.8

^a^ The non-equivalent nitro-groups in BNFF-1 are denoted with (I) and (II) and correspond to Figure 2. ^b^ Reaction energies are shown in parentheses. For the barrierless reactions of the homolytic bond cleavage, the reaction energy coincides with the bond dissociation energy. ^c^ The NO loss is a secondary reaction step following the CONO isomerization.

### 3.1. The Homolytic C-NO_2_ Break

The homolytic C-NO_2_ break was simulated following the standard Equation (1):

X-(C_2_N_2_O)_3_-NO_2_ → X-(C_2_N_2_O)_3_^●^ + NO_2_^●^ (1)

where X stands for a nitro-group in BNFF-1 and an amino-group in ANFF-1.

According to our calculations, the reaction barrier in BNFF-1 requires 57.6–63.1 kcal/mol depending on the computational method used, indicating a slightly higher activation barrier for the nitro-group (II) than for the nitro-group (I), shown in Table 1. The difference in obtained energies is however very small and does not allow for a definite discrimination of one nitro-group vs another. All three DFT functionals, PBE, M06, and B3LYP give consistent energies, which are very close to each other, with the M06 value being the highest (63.1 kcal/mol for nitro-group (I) and 65.6 for the nitro-group (II)), the B3LYP energy being the lowest (57.6 and 60.0 kcal/mol, respectively), and PBE energy falling right in the middle (61.1 and 62.2 kcal/mol, respectively). The similar energies obtained for the ANFF-1, 59.5–61.5 kcal/mol are even closer to each other, showing practically no dispersion. The estimated pre-exponential factors point towards the NO_2_ loss as the fastest overall initiation reaction among all investigated mechanisms (Table 1). 

Experimental and theoretical data on BNFF-1 and ANFF-1 are limited at the time being, therefore we will link our results to existing relevant literature on other nitro-compounds and general trends reported in earlier studies [44,45,46]. The calculated activation energies of the C–NO_2_ homolysis of ANFF-1, 59.5–61.1 kcal/mol, and BNFF-1, 57.6–63.1 kcal/mol, rest at the lower end of the range of the typical dissociation energies of C–NO_2_ bonds, 61–70 kcal/mol, determined for nitrofurazan (56.8 kcal/mol [47]), C-nitro derivatives of triazole (~67 kcal/mol [48]), a wide variety of nitroaromatics [44], including TATB (59–64 kcal/mol [45,49], ~70 kcal/mol [50,51]), DADNE (58–67.0 kcal/mol [52], ~70 kcal/mol [53]), and a series of related aminotrinitrobenzene compounds [45,46]. 

### 3.2. The CONO-Isomerization Precursor of the NO Loss

The nitro-nitrite isomerization proceeds via a pseudo rotation of a nitro group accompanied by breaking the C-NO_2_ bond and creating the C-ONO bond [Equation (2)]:

X-(C_2_N_2_O)_3_-NO_2_ → X-(C_2_N_2_O)_3_-ONO (2)

**Figure 4:**
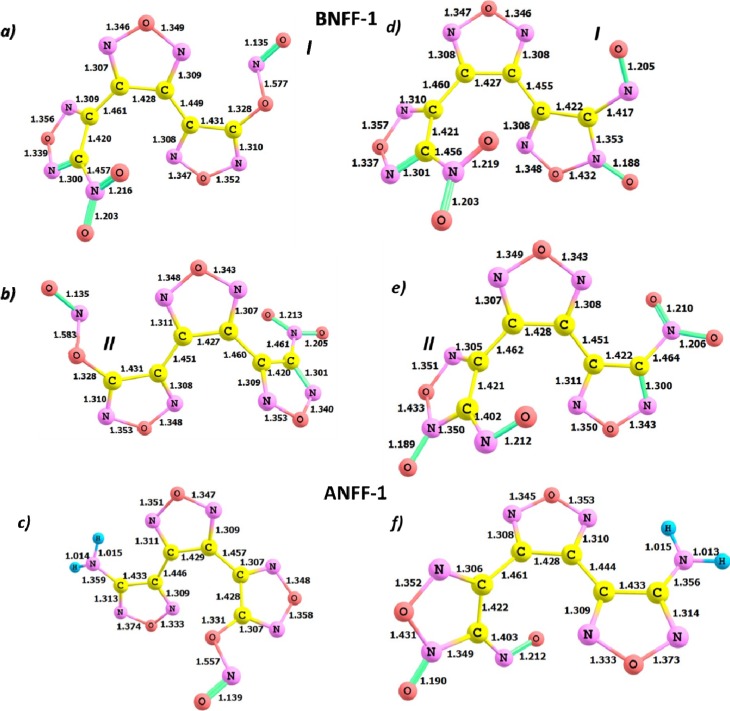
The structures of (**a**–**c**) nitrite-isomers and (**d**–**f**) nitroso-furoxan isomers of BNFF-1 and ANFF-1. The depicted bond distances (Å) correspond to the M06 level of theory.

**Figure 5:**
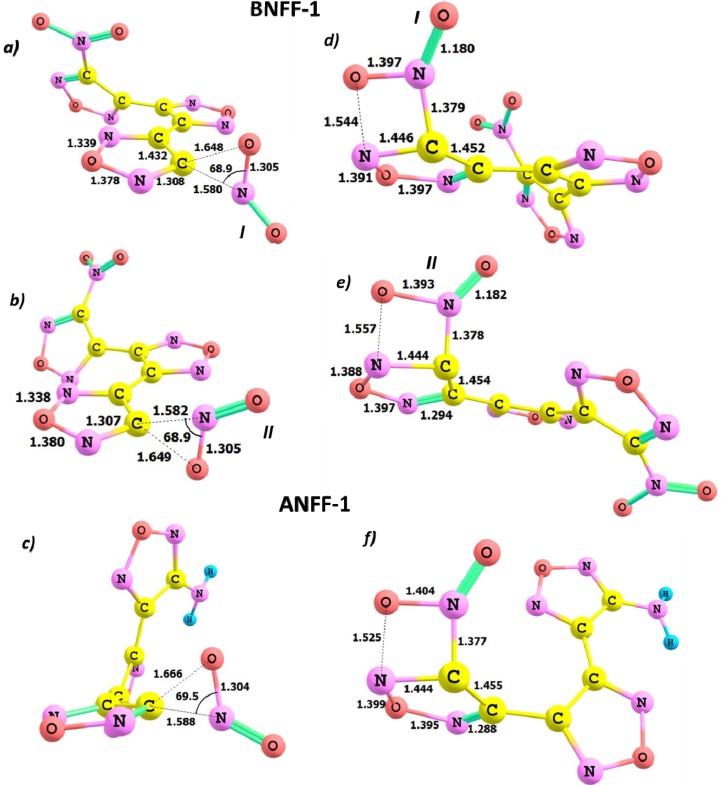
The structures of transition states of (**a**–**c**) the CONO rearrangement and (**d**–**f**) the O-transfer reaction pathways in BNFF-1 and ANFF-1. The bond distances (Å) are obtained with M06 functional.

The formed nitrite isomers of BNFF-1 and ANFF-1 are shown in Figure 4a–c and the transitions states are displayed in Figure 5. The CONO-isomerization in BNFF-1 appears to require a somewhat lower activation barrier (by 4–15 kcal/mol depending on the method) than the NO_2_ loss reaction pathway (Table 1), and the NO loss needs an even lower energy of 19.8–30.9 kcal/mol. Besides, the CONO-isomerization is an exothermic reaction, releasing heat [54] of 12.8–14.6 kcal/mol (Table 1). 

Although the nitro-group (I) shows a slightly smaller activation barrier and a slightly higher reaction heat than the nitro-group (II), the observed difference is too small to claim a distinction. In the meantime, the dispersion of energies resulting from different functionals deserves some attention. PBE gives the lowest energy, 46.1 kcal/mol, for the CONO-isomerization *versus* 53.2 kcal/mol obtained with both M06 and B3LYP. PBE also yields the highest energy for the NO loss, 30.9 kcal/mol, *versus* 25.0 kcal/mol obtained with M06 and 19.8 kcal/mol obtained with B3LYP (see the reaction 2 in Table 1). Given that M06 and B3LYP produce the same value of 53.2 kcal/mol and taking into account that PBE tends to underestimate molecular rearrangements [36], the difference of 4 to 10 kcal/mol between the CONO-isomer formation and the NO_2_ loss reactions appears more realistic than 4–15 kcal/mol. 

Similar results are obtained for the ANFF-1 molecule with close activation barriers of the CONO-isomerization stage ranging from 51.9 to 60.3 kcal/mol, an appreciable difference in energies of the NO-loss stage (18.6–29.8 kcal/mol), and the reaction heat of 8.2–10.7 kcal/mol.

The calculated activation energies of the nitro-nitrite isomerization of BNFF-1 and ANFF-1 are consistent with the barrier of the CONO formation in DADNE (66.4 kcal/mol [52], 59 kcal/mol [55]) and nitromethane, (55.5 kcal/mol, estimated from infrared multiphoton dissociation experiments [56], and 51.7 kcal/mol, obtained from *ab initio* calculations [57]). The similar rearrangement in nitroethylene was found to require about 57.9 kcal/mol [58]. Close barriers were also obtained in C-nitro isomers of triazole (60.1–65.2, 63.5 kcal/mol [48]) and nitroaromatics, nitrobenzene (61.1 kcal/mol [59], 63.7 kcal/mol [60]) and trinitrotoluene (54.9 kcal/mol [61]). The BNFF-1 and ANFF-1’s behavior (Table 1) resembles the nitroethylene’s decomposition trend (with CONO-isomerization requiring ~15 kcal/mol less than the energy needed to cleave the C–NO_2_ bond) [58] and somewhat differs from that of DADNE, which exhibits nearly isoenergetic reactions of the nitro-nitrite isomerization and the C-NO_2_ cleavage [52]. Another difference is that the CONO isomerization in nitrobenzene is an endothermic reaction (1.1 kcal/mol [59] and 4.0 kcal/mol [60]) whereas in DADNE it is only weakly exothermic with the energy gain of 4 kcal/mol [52,55] *vs*. relatively higher heat produced in BNFF-1 (12.8–14.6 kcal/mol) and in ANFF-1 (8.2–10.7 kcal/mol, Table 1), similar to trinitrotoluene (8.1 kcal/mol [61]).

**Figure 6:**
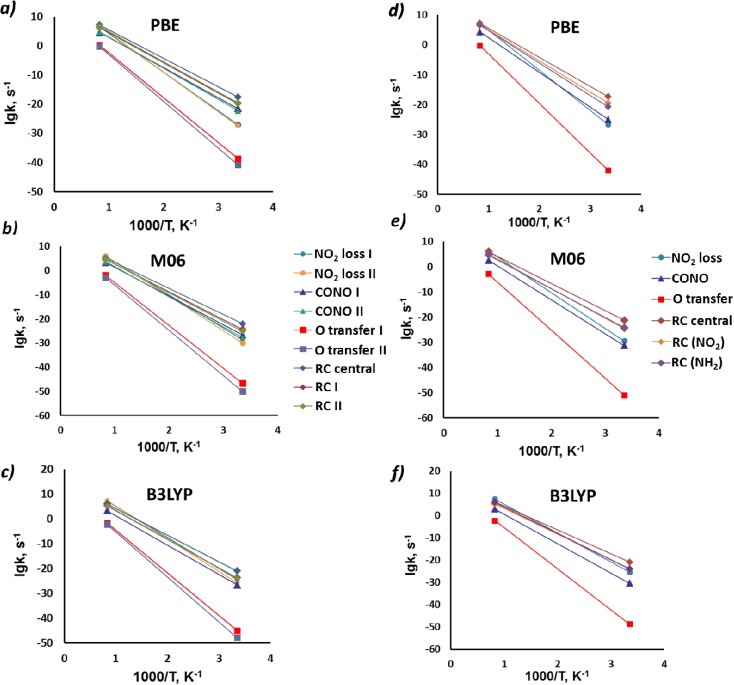
Reaction rates of the simulated decomposition channels of BNFF-1 (***a***–***c***) and ANFF-1 (***d***–***f***) molecules.


An analysis of the kinetic parameters, generated by both PBE and M06, predicts a competition between the CONO formation and the C-NO_2_ fission in BNFF-1 over a wide temperature range while kinetics obtained from B3LYP suggests that the C-NO_2_ homolysis is favored over all other probed mechanisms (Table 1 and Figure 6). Unlike BNFF-1, only PBE supports the CONO-isomerization while both M06 and B3LYP prefer the C-NO_2_ break in the initiation of decomposition of ANFF-1. Hence, the CONO preference achieved from the energetic considerations is practically negated once the kinetics is taken into account. 

We emphasize here that energetic considerations should be coupled with kinetics to understand the thermal decomposition, especially, when several chemical pathways co-exist. Thus, the exothermic nitro-nitrite isomerization and the NO loss require a relatively low energy but the pre-exponential factor of this dissociation reaction is estimated to be much smaller than that of the NO_2_ loss (Table 1). Hence, our analysis suggests that the C-NO_2_ fission is the fastest dissociation process while the NO loss contributes to decomposition as a slow background reaction. Interestingly, neither of these two reactions is the dominant initiation mechanism in the decomposition of BNFF-1 and ANFF-1 (*vide infra*).

### 3.3. Oxygen Transfer

Oxygen transfer, another type of isomerization, results in a formation of nitroso-furoxan isomers of BNFF-1 and ANFF-1 displayed in Figure 4d–f [Equation (3)]. The O-transfer mechanisms in both molecules have significantly higher activation barriers (~70–80 kcal/mol in BNFF-1 and ~75–89 kcal/mol in ANFF-1) than the corresponding homolytic cleavage of the C-NO_2_ bonds and the CONO-isomerization reactions and exhibit fairly low pre-exponential factors (reaction 3 in Table 1 and Figure 3). The difference obtained for the different nitro-groups is again small. Hence, this reaction should be ruled out as a possible candidate to initiate the decomposition of either BNFF-1 or ANFF-1.

X-(C_2_N_2_O)_3_-NO_2_ → X-(C_2_N_2_O)_2_-C(NO)OCN(O) (3)

We do not consider a hydrogen transfer here for two main reasons: (1) the hydrogen migration is strongly dependent upon the molecular environment (and it is therefore unreliable to model it for molecules) and (2) the HONO reaction typically represents only a secondary isomerization pathway in nitro-arenes [52,62].

### 3.4. The Homolytic C-C Break

The homolytic fission of the C-C bonds in the molecules [Equation 4(a,b)] are also energetically unfavorable reaction pathways with the calculated dissociation energies of ~121–125 kcal/mol in BNFF-1 and ~123–128 kcal/mol in ANFF-1, which are much higher than the corresponding energies required for breaking the C-N bonds (reactions 4, Table 1 and Figure 3). These estimates agree with the C-C dissociation energy of a DADNE molecule (120.2 kcal/mol) [52].

X-(C_2_N_2_O)_3_-NO_2_ → X-(C_2_N_2_O)_2_^●^ + ^●^(C_2_N_2_O)-NO_2_ (4a)

X-(C_2_N_2_O)_3_-NO_2_ → X-(C_2_N_2_O)^●^ + ^●^(C_2_N_2_O)_2_-NO_2_ (4b)

The difference in energy between splitting off of the peripheral nitro-furzan rings in BNFF-1 does not exceed 2 kcal/mol. The distinction between the nitro-furazan ring’s loss and amino-furazan ring loss is comparable, ~4 kcal/mol, signifying a measure of the substitution effect (reaction 4, Table 1). Hence, the splitting off the rings would not contribute to the initial stages of the overall decomposition of either molecule. 

### 3.5. A Heterocyclic Ring Cleavage (RC)

Opening of the heterocyclic ring may be in principle achieved through breaking one of the ring’s bonds (see, for example, ref. [47]). We explored concerted fragmentation mechanisms of BNFF-1 and ANFF-1 molecules (path 5 in Figure 3 and reaction 5 in Table 1) in which a cleavage of the central furazan ring [Equation 5(a)] or one of the peripheral rings [Equation 5(b),(c)] proceeds through a simultaneous fission of the N-O and C-C bonds:

X-(C_2_N_2_O)_3_-NO_2_ → X-(C_2_N_2_O)CN + CNO-(C_2_N_2_O)-NO_2_ (5a)

X-(C_2_N_2_O)_3_-NO_2_ → XCNO + CN-(C_2_N_2_O)_2_-NO_2_ (5b)

X-(C_2_N_2_O)_3_-NO_2_ → X-(C_2_N_2_O)_2_-CN + CNO-NO_2_ (5c)

**Figure 7:**
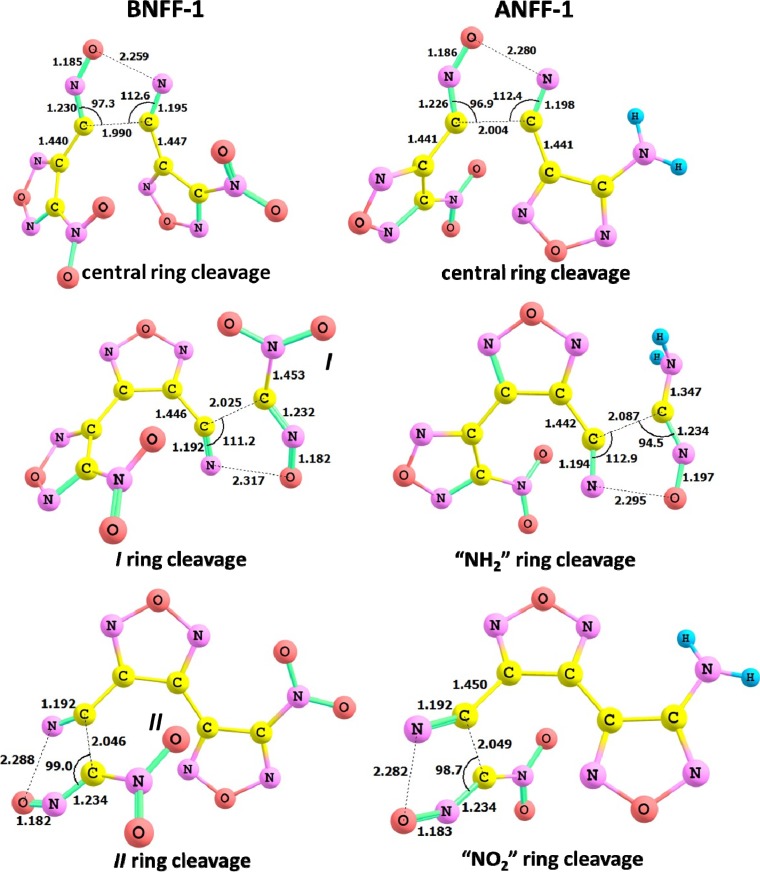
The structures of transition states for ring cleavage reactions in BNFF-1 and ANFF-1. The bond distances (Å) are obtained with M06 functional.

The transition state structures, shown in Figure 7, indicate an elongation of N-O (~1 Å) and C-C (~0.6 Å) bonds in the furazan rings. The calculations reveal that the ring cleavage reactions in BNFF-1 require 42.9–51.1 kcal/mol of energy. The endothermic cleavage of the central ring (Figure 3) in BNFF-1 requires 42.9–47.6 kcal/mol, with the lowest barrier (42.9 kcal/mol) obtained with PBE and very close barriers of 47.6 and 47.5 kcal/mol obtained with M06 and B3LYP, respectively. This energy is slightly (~3 kcal/mol) lower than the barriers for the opening of peripheral rings (reaction 5 in Table 1). An energy dispersion obtained with different functionals is ~5 kcal/mol, with a systematic difference observed between PBE and hybrid methods and almost identical energies received from M06 and B3LYP. The energy difference between outer nitro-furazan rings does not exceed 1 kcal/mol. 

Consistent activation barriers (42.7–55.6 kcal/mol) and reaction energies are obtained for ANFF-1. The same trends are seen: the central ring opening needs a lower energy than the peripheral rings, and all three methods produce corresponding energies with the lowest barrier (42.7 kcal/mol) delivered by PBE and nearly identical barriers (47.6 and 47.1 kcal/mol obtained from M06 and B3LYP). The difference in energy between cleavage of the nitro-furazan ring and the amino-furazan ring is negligible. 

Most importantly, the concerted ring opening reactions exhibit the lowest activation barriers among all mechanisms explored in both molecules, BNFF-1 and in ANFF-1. These values agree with the activation energy of 42.3 kcal/mol obtained from differential scanning calorimetry measurements of BNFF [21]. Pre-exponential factors (log(*A*/^s−1^) ~15, Table 1) of the ring cleavage mechanisms are still lower than the NO_2_ loss but visibly higher than the CONO isomerization. The estimated reaction constant is consistent with the log(*A*/^s−1^) = 13.68, reported for BNFF [21]. Kinetic behavior is illustrated in Figure 6, which shows that the fragmentation through the central ring opening dominates the decomposition initiation in both molecules at low and moderate temperatures (regardless of the computational method used). The NO_2_ loss, while being the fastest reaction, prevails only at high temperatures. The exothermic CONO-isomerization pathway makes a modest contribution to decomposition at the earliest stages. 

## 4. Comparing BNFF-1 and ANFF-1

Next, let us turn to the difference between the BNFF-1 and ANFF-1 decomposition processes, or discuss the substitution effect here. In both molecules, the cleavage of the central ring seems to be the most favorable dissociation process from an energetic point of view (42.9–47.6 kcal/mol in BNFF-1 *vs*. 42.7–47.6 kcal/mol in ANFF-1), with a negligible difference between ANFF-1 and BNFF-1. The energy of the C-NO_2_ bond dissociation in ANFF-1 (61.1 delivered by PBE, 63.1 by M06, and 57.6 kcal/mol by B3LYP) also shows only a small change as compared to BNFF-1 (61.3, 61.5, and 59.5 kcal/mol, respectively). The C-NO_2_ bond pre-exponential factors evidence that the detachment of NO_2_ from BNFF-1 and ANFF-1 will proceed at comparable rates and faster than other reactions. At the same time, dissociation energies coupled with kinetic parameters point towards the ring opening as a dominating dissociation process at low and moderate temperatures while the C-NO_2_ homolysis becomes important at high temperatures. 

The most notable effect of the BNFF-1’s nitro group replacement with the ANFF-1’s amino group appears in the barrier heights and reaction energies of the CONO decomposition channel. The CONO-isomerization of ANFF-1 (51.9 kcal/mol found by PBE, 59.1 kcal/mol by B3LYP, and 60.3 kcal/mol by M06) requires ~6 kcal/mol higher energy in comparison to BNFF-1 (46.1 kcal/mol obtained by PBE and 53.2 kcal/mol by M06 and B3LYP) while the exothermicity of this pathway is reduced by ~4 kcal/mol (Table 1). Due to a somewhat higher activation barrier and somewhat lower reaction heat of ANFF-1 than those of BNFF-1, the contribution of the slow exothermic CONO-isomerization reaction in BNFF-1 is expected to be more pronounced as compared to ANFF-1. However, we suggest that this process has only a minor effect on the overall thermal dissociation and hence the presence of nitro- or amino-groups in these heterocyclic molecules does not make a significant difference.

## 5. Summary and Conclusions

Modeling of candidate decomposition channels of new energetic compounds BNFF-1 and ANFF-1 was performed by means of density functional theory combined with transition state theory. The obtained activation barriers, reaction energies, nature of the transition states, pre-exponential factors, and reaction rates were analyzed for five plausible decomposition channels, including the homolytic NO_2_ loss, step-wise NO loss, switching of N-O bonds through the oxygen transfer, splitting off of the heterocyclic rings by cleavage of the C-C bond, and concerted opening of the heterocyclic rings. A careful evaluation revealed that the cleavage of the central furazan ring dominates the overall decomposition initiation of both BNFF-1 and ANFF-1 molecules, and breaking the peripheral rings requires only slightly higher energy. The obtained decomposition activation barriers of both BNFF-1 and ANFF-1 agree with DSC measurements of BNFF. The next important decomposition mechanism is the homolytic C-NO_2_ bond cleavage. Despite being the fastest reaction, the C-NO_2_ homolysis prevails only at high temperatures due to a notably higher activation barrier than that of the ring opening process. The two-step NO loss in both molecules, while it exhibits a somewhat higher activation barrier than the ring opening reactions and may co-exists with the primary dissociation channel, proceeds at a much lower rate. 

Thus, our calculations established that the decomposition scenario in heterocyclic nitro-furazan-based molecules differs from known nitro-arenes. Unlike nitro-arenes, the thermal decomposition in BNFF analogs is strongly affected by the presence of heterocyclic rings and the ring’s opening reactions trigger the overall bond dissociation. A further development of the dissociation processes is defined by the interplay of the slow exothermic CONO-isomerization and the fast C-NO_2_ homolysis reactions. While the activation barrier of the NO loss requires a lower energy than the NO_2_ detachment, the reaction kinetics happened to be orders of magnitude slower for the CONO-isomerization than for C-NO_2_ homolysis.

The substitution of one nitro group in BNFF-1 with the amino group in ANFF-1 does not affect the ring opening reactions, negligibly decreases the C-NO_2_ bond strength, tends to increase the CONO-isomerization activation barrier and to reduce the reaction heat. 

The analyzed decomposition pathways provide valuable insight on the dissociation of bonds in these complex molecules. However the derived conclusions cannot be simply extrapolated to predict materials’ behavior. The next step in this research is sophisticated solid-state periodic calculations involving modeling of similar chemical reactions in the crystalline environment [4,5,13,14] and on surfaces [13,42,43,63]. In addition, the effect of crystallographic and electronic defects [15,52,62] has to be taken into account as, at least for DADNE, it was discovered that the shear-strain [4,5,14] and other imperfections [12] severely affect the decomposition process by reducing the decomposition barriers and/or enhancing exothermicity [52] and hence decrease sensitivity of the material to initiation of detonation.
